# Improving Knowledge about Children’s Environmental Health in Northwest China

**DOI:** 10.3390/ijerph13010080

**Published:** 2015-12-25

**Authors:** Jingping Niu, Qingshan Qu, Juansheng Li, Xingrong Liu, Benzhong Zhang, Zhilan Li, Guowu Ding, Yingbiao Sun, Yanrong Shi, Yaxiong Wan, Xiaobin Hu, Lung-Chi Chen, Alan Mendelsohn, Yu Chen, Leonardo Trasande

**Affiliations:** 1 Departments of Environmental Medicine, Lanzhou University School of Public Health, 199 Donggang Xi Lu, Lanzhou, Gansu 730000, China; lijsh@lzu.edu.cn (J.L.); liuxr@lzu.edu.cn (X.L.); zhangbzh@lzu.edu.cn (B.Z.); lizhl@lzu.edu.cn (Z.L.); dinggw@lzu.edu.cn (G.D.); sunyb@lzu.edu.cn (Y.S.); shiyr13@lzu.edu.cn (Y.S.); wanyx@lzu.edu.cn (Y.W.); huxiaobin@lzu.edu.cn (X.H.); Yu.Chen@nyumc.org (Y.C.); 2 Department of Pediatrics, New York University School of Medicine, 227 East 30th Street, Rm 735, New York, NY 10016, USA; Qingshan.Qu@nyumc.org (Q.Q.); lung-chi.chen@nyumc.org (L.-C.C.); 3 Department of Population Health, New York University School of Medicine/Bellevue Hospital Center, New York, NY 10016, USA; Alan.Mendelsohn@nyumc.org; 4 New York University School of Medicine, New York, NY 10016, USA; 5 Wagner School of Public Service, New York University, New York, NY 10012, USA; 6 Department of Nutrition, Food & Public Health, Steinhardt School of Culture, Education and Human Development, New York University, New York, NY 10003, USA

**Keywords:** children’s environmental health, policy making, air pollution, educational intervention

## Abstract

The main purpose of this study was to identify policy maker opinions and attitudes towards children’s environmental health (CEH), potential barriers to child-specific protective legislation and implementation in northwest China, and evaluate knowledge and attitudes about CEH before and after an educational conference. We conducted seventy-two interviews with regional officials, researchers and non-governmental organization representatives from five provinces, and surveyed participants (forty-seven) before and after an educational conference in northwest China about CEH. Interviews identified general consensus among participants of the adverse effects of air pollution on children, yet few participants knew of policies to protect them. Barriers identified included limited funding and enforcement, weak regional governments and absence of child-specific policy-making. After the conference, substantially greater self-efficacy was identified for lead, mercury, air pollution and polychlorinated biphenyls (+0.57–0.72 on a 1–5 Likert scale, *p* = 0.002–0.013), and the scientific knowledge for the role of environment in children’s health (+0.58, *p* = 0.015), and health care provider control (+0.52, *p* = 0.025) were rated more strongly. We conclude that policy makers in Northwest China appreciate that children are uniquely vulnerable, though additional regulations are needed to account for that vulnerability. Further research should examine effectiveness of the intervention on a larger scale and scope, and evaluate the usefulness of such interventions in translating research into improved care/reduced exposure to environmental hazards.

## 1. Introduction

Accelerated economic growth in the People’s Republic of China (PRC) has rapidly increased early life exposures to environmental hazards. Coal consumption and production have quadrupled, increasing mercury emissions, with fish and rice contamination with methylmercury raising concerns about adverse effects on early neurodevelopment [1,2,3]. Lead emissions from lead acid battery production, mining, and smelting have produced outbreaks of childhood lead poisoning [4,5,6], and one-third of Chinese children may have blood lead ≥10 µg/dL [7]. Industrialization in China was most intense in the eastern part during the 1980s and 1990s. Since 2000, rapid transformation has ensued especially in the northwest provinces of China as part of a new state policy, known as China’s Western Development strategy [8].

Children have increased vulnerability to many common environmental toxins. This is because of specific exposure pathways such as dust and soil ingestions [9] based on their distinctive activity patterns and higher body weight normalized intakes [10] compared to adults. These factors, in addition to the increased susceptibility of the developing organs (e.g., immaturity of several detoxification pathways) [11] contribute to the unique vulnerability of children to environmental toxins.

In a previous study, we conducted a survey of child health providers (695 surveys analyzed) from five provinces in northwest PRC (Gansu, Shaanxi, Xinjiang, Qinghai, and Ningxia) to assess knowledge about environmental hazards, and self-efficacy in identifying, assessing and managing these exposures and their consequences [12]. Though self-efficacy reported with managing lead, pesticide, air pollution, mercury, mold and polychlorinated biphenyl exposures were generally modest (2.14–2.52 mean on a 1–5 Likert scale), the role of environment in health was reported to be strong (mean 4.31) and assessment of exposures using environmental history was also rated highly (mean 3.88). Among the providers, 92.5% reported patients affected by environmental exposures, with 14% of providers reporting >20 affected patients in their practice.

Given the substantial impacts of environmental exposure on child health that providers have documented in Northwest PRC, a significant opportunity exists for policy change to prevent disease and disability. Policy maker attitudes have rarely been assessed in a rigorous way in the developing world [13]. Educational interventions have the opportunity to increase knowledge both in health care providers and policy makers, yet these have not been assessed in the context of a rapidly developing country like PRC.

This report describes findings of interviews performed with seventy-two regional officials, researchers and non-governmental organization representatives from the five provinces in Northwest PRC, to identify their opinions and attitudes towards children’s environmental health, as well as potential barriers to child-specific protective legislation and implementation. We also describe results of surveys administered to child health providers, community stakeholders and decision makers. This was used to evaluate knowledge, attitudes and beliefs about children’s environmental health before and after an educational conference in Northwest PRC.

## 2. Methods

### 2.1. Interviews with Regional Officials, Researchers and Non-Governmental Officials

Two of the investigators (Xingrong Liu, Juansheng Li) identified a sample of regional and national officials, researchers and non-governmental organization representatives, to represent a range of experiences and attitudes towards children’s environmental health in the region and to reflect a broader range of potential barriers to child-specific protective legislation and implementation. The approach followed that used to assess children’s environmental health policy in Mexico in which one of the authors (Leonardo Trasande) was involved [13]. Potential participants were identified based on their role in environmental and health policy. Snowballing techniques were also used, in which study subjects identified additional subjects who would add meaningful insights and perspectives [14].

Interview guidelines, surveys and questionnaires were translated into Mandarin by native speakers and back-translated to confirm accuracy. Prior to commencing the interviews, an annotated outline was developed along with interview guidelines focusing on eleven questions and three major themes (Beliefs and Attitudes, Current Activities, and Barriers and Solutions. Table 1).

**Table 1:** Overarching questions and themes for policy interviews.

**Beliefs and Attitudes**
1. What do you know/believe about impacts of air pollution on health?
2. What do you know/believe about impacts of air pollution on health in children?
3. What is the role of research (environmental and public health research) in informing policy to protect children?
4. Who are the key decision makers in protecting children from air pollution?
**Current Activities**
5. What research is being done to protect children from air pollution?
6. What policies exist to protect children from air pollution?
7. What are health care providers doing to protect children from air pollution?
**Barriers and Solutions**
8. What barriers are there to protecting children from air pollution?
9. What are the gaps in policies to protect children from air pollution?
10. Who are the key strategic players in protecting children from air pollution?
11. What are potential recommendations to protect children from air pollution?

Relevant international, national and regional guidelines were used to develop content for structured interviews around these themes and individual questions [15,16]. We conducted an extensive literature review before developing the interviews and adapted the methodology described in the WHO Children’s Environmental Health Survey on Child Environmental Health Awareness of Health Care Professionals [17]. For the analysis of our data we also relied on the Partnerships for Environmental Public Health Evaluation Metrics Manual which provides extensive guidelines for planning and evaluation instruments [18].

Each interviewee was approached separately, and all interviews were conducted privately by investigators who have no direct working relationship with the interviewees. Interviews were recorded and transcribed with permission, and responses were sorted according to major questions. Inductive and deductive analyses of the transcripts [19,20] focused on identifiable patterns of quotes and statements. These analyses were used to develop overarching themes that captured approaches to policy making and governance in Northwest PRC around children’s environmental health [21]. We coded responses as belonging to one or more of the major themes [15,16].

### 2.2. Pre- and Post-Conference Evaluation of Attitudes, Beliefs and Behaviors

Findings from the policy interviews as well as the previously described health care provider surveys [12] informed the formulation of an agenda for a two-day regional conference. Child health providers, community stakeholders and decision makers were invited to attend, and encouraged to ask others to join. The agenda was focused on outdoor air pollution, with additional sessions providing context for other environmental exposures to which children are vulnerable. Learning provided in the didactic sessions was complemented by small group discussions that permitted exploration of individual perspectives that would not otherwise be readily identified. The conference was held at Lanzhou University School of Public Health from 26 to 29 September 2013. Participation was free, and scholarships were provided to support travel to and lodging in Lanzhou.

Pre-conference surveys were divided into three parts. The first part evaluated beliefs and attitudes towards children’s environmental health, as well as knowledge regarding lead, pesticide, air pollution, mercury, mold and polychlorinated biphenyl exposures. These questions asked providers to evaluate their perceptions about the role of the environment in children’s health, the sufficiency and adequacy of the scientific evidence for the impact of the environment on children, whether environmentally mediated disease in children was increasing, and the ability of child health providers, industry and government to control environmental health hazards. They were also asked to evaluate whether policies in place were sufficient to protect children. Some of these questions were identical to those used in the health care provider surveys, in order to ensure comparability [12]. Participants rated their agreement with this series of belief statements on a Likert scale of 1–5, from “strongly disagree” to “strongly agree”.

The second part asked participants to identify key decision makers and strategic players in protecting children from air pollution, policies that exist to protect children, activities health care providers are taking to protect children, barriers to protecting children, and gaps in policies to protect children. Participants were permitted to select multiple options. The third part asked demographic information including age, gender, provincial activity, and type of work (health care provider, government official or other).

Questionnaires administered at the end of the conference reprised the first part of the questionnaire to assess interval change in beliefs and attitudes. They also reprised demographic questions to facilitate matching of pre- and post-conference questionnaires. Finally, two questions asked whether the conference provided important information regarding children and environmental hazards, and whether it provided information that will/will not change practice/policy making.

Statistical analyses of the survey data were performed using Stata 13.0 (Stata, College Station, TX, USA). In addition to descriptive analyses, Student t-tests were used to compare means of Likert scales among participants before the conference to those after the conference.

### 2.3. Human Subjects

This research involving human subjects was performed in accordance with the Declaration of Helsinki, and interview guidelines, surveys and questionnaires were approved by the New York University School of Medicine and Lanzhou University School of Public Health Institutional Review Boards, with a waiver of signed consent.

## 3. Results and Discussion

### 3.1. Results

#### 3.1.1. Interviews with Regional Officials, Researchers and Non-Governmental Officials

We completed interviews of seventy-two regional officials, researchers and non-governmental organization representatives (Table 2). Participation rate was >95%.

**Table 2:** Policy interview characteristics.

Characteristic	*N* (%)
Province	–
Gansu	24 (33%)
Shanxi	13 (18%)
Ningxia	14 (19%)
Qinghai	10 (14%)
Xinjiang	11 (15%)
Agency/Organization	–
Environmental Protection Bureau	18 (25%)
Health Department	31 (43%)
Medical Service	21 (29%)
Nongovernmental Organization	2 (3%)
Rank	–
Commissioner of Provincial Department of Health or Bureau of Environmental Protection	25 (35%)
Deputy/Mid-Level with Oversight of Provincial Department of Health or Bureau of Environmental Protection	12 (17%)
Staff of Provincial Department of Health or Bureau of Environmental Protection	21 (29%)
Director/President of Hospital	14 (19%)
Medical Service Provider at Hospital	5 (7%)

These interviews identified general consensus of the impact of air pollution on children’s health and their vulnerability. While consensus existed about the role of government in protecting children from air pollution exposures, few knew of policies to protect children from these exposures. A diverse array of barriers were identified that could potentially protect children from air pollution exposure—these included lack of funding, lack of enforcement of existing policies, weak regional governments and lack of proven and targeted policies aimed at protecting children. Industry was not identified as having a role in resolving issues about children’s exposure to air pollution, yet industrial processes are important sources of environmental pollution and exposure. This exclusion may have resulted from the general manner in which we posed the question, without suggesting potential responses, such as specific governmental agencies, non-governmental organizations, or industries. It should also be noted that all interviewees were from either environmental or health related fields and did not have industry experience, and they may have therefore overlooked the role of industry in their response. While additional research was also cited as necessary, the primary suggestions were for additional government regulation and education to prevent associated health effects.

#### 3.1.2. Pre- and Post-Conference Evaluation of Attitudes, Beliefs and Behaviors

Forty-seven participants, including health care providers and local Health and Environmental Protection Agency officials from each of the five provinces participated in the conference, and completed pre-conference surveys, of whom forty-two (89%) responded to the post-conference questionnaire. Participants were predominantly female (78.6%), with the average age among participants of 38.6 years (Table 3).

Surveys of participants before the conference (Table 4) revealed similar agreement of the role of environment in health (mean 4.52 on a 1–5 Likert scale), and weak self-efficacy in managing common exposures among providers for lead, mercury and air pollution exposures (mean 2.19–2.81).

Scientific evidence for the impact of environment on children (mean 3.10) was also rated more modestly. Health care provider control over hazards was rated much more modestly (mean 2.81) than government and industry control (means 4.13–4.21). Participants predominantly identified national (63%) and provincial (46%) environment and national health (44%) officials as key decision makers in protecting children from air pollution (Table 5).

Few participants identified policies to protect children from air pollution, with just over a third (38%) identifying national environmental regulations protecting children and just over a fourth (29%) identifying national health regulations with this purpose.

**Table 3:** Description of respondents and their practices.

Characteristic	*N* (%)
Age, mean ± SD	38.6 ± 9.8
Sex	
Male	9 (21.4)
Female	33 (78.6)
Practice type	
Primary health care	9 (21.4)
Specialty health care	16 (42.8)
Public health/other government official	17 (45.2)
Province	
Gansu	12 (28.6)
Xinjiang	7 (16.7)
Shaanxi	8 (19.0)
Qinghai	10 (23.8)
Ningxia	5 (11.9)

**Table 4:** Conference participant self-reported beliefs and self-efficacy regarding environmental health.

**Belief Statements (*n* = 47)**	**Mean ± SD**
The role of environmental health impacts on children is of little importance (1) → of great importance (5)	4.52 ± 0.74
Scientific evidence for the impact of the environment of children is not sufficient (1) → sufficient (5)	3.10 ± 1.08
The magnitude of children’s environmental related-illnesses is decreasing (1) → increasing (5)	4.54 ± 0.77
The control child health providers have over environmental health hazards is minimal (1) → maximal (5)	2.81 ± 1.21
The control government has over environmental health hazards is minimal (1) → maximal (5)	4.17 ± 1.19
The control industry has over environmental health hazards is minimal (1) → maximal (5)	4.13 ± 1.10
Assessing environmental exposures through history-taking in pediatric practice is of little importance (1) → of great importance (5)	4.21 ± 0.86
Conducting an environmental health history on all my patients (1) takes up too much time → does not take up too much time (5)	1.89 ± 0.83
**Self-Efficacy Statements (*n* = 47)**	**Mean ± SD**
How would you rate your knowledge regarding:	–
Lead exposure	2.56 ±1.03
Pesticide exposure	2.73 ± 1.14
Air pollution exposure	2.19 ± 0.89
Mercury exposure	2.54 ± 0.95
Mold exposure	2.81 ± 1.12
PCB exposure	2.35 ± 0.84

**Table 5:** Conference participant perspectives about key decision makers and opportunities to protect children from air pollution.

Question	*N* (%)
*Who are the key decision makers in protecting children from air pollution?*	–
Health care providers	8 (17%)
Provincial environmental officials	22 (46%)
Provincial health officials	13 (27%)
Local health officials	7 (15%)
Local environmental officials	11 (23%)
National health officials	21 (44%)
National environmental officials	30 (63%)
Industry	15 (31%)
Researchers	7 (15%)
*What policies exist to protect children from air pollution?*	–
Provincial environmental regulations	8 (17%)
Provincial health regulations	4 (8%)
Local health regulations	2 (4%)
Local environmental regulations	5 (10%)
National health regulations	14 (29%)
National environmental regulations	18 (38%)
*What are health care providers doing to protect children from air pollution?*	–
Provide educational materials	16 (33%)
Assess environmental exposures	15 (31%)
Communicate prevention measures to families	22 (46%)
Treat patients with conditions of environmental origin	19 (40%)
*What barriers are there to protecting children from air pollution?*	–
Industry profits	33 (69%)
Lack of governmental regulation	39 (81%)
Focus of government on economic growth	28 (58%)
Lack of medical knowledge	18 (38%)
Lack of research	19 (40%)
Lack of public awareness	33 (69%)
*What are the gaps in policies to protect children from air pollution?*	–
Lack of policies	25 (52%)
Weak enforcement	32 (67%)
Lack of research	20 (42%)
Policies do not address children	26 (54%)
*Who are the key strategic players in protecting children from air pollution?*	–
Industry	14 (29%)
Provincial environmental agency	31 (65%)
Provincial health agency	23 (48%)
Local health agency	10 (21%)
Local environmental agency	14 (29%)
National health agency	24 (50%)
National environmental agency	28 (58%)
Medical provider	7 (15%)
Researchers	7 (15%)

Barriers to protecting children included lack of governmental regulation (81%), a focus on economic growth by government (58%), followed by lack of public awareness and industry profits (both 69%) and lack of medical knowledge (38%). Weak enforcement (67%) was deemed to be the chief barrier to policies to protect children, though lack of policies and research both were rated as gaps by a majority. National and provincial environmental and health agencies were identified as key strategic players in protecting children (48%–65%) with many fewer respondents identifying industry, local agencies, medical providers or researchers (<30%) as key strategic players.

After the conference, substantially greater self-efficacy was identified for lead, mercury, air pollution and polychlorinated biphenyls (+0.57–0.72, *p* = 0.002–0.013), and both scientific knowledge for the role of environment in children’s health (+0.58, *p* = 0.015), and health care provider control (+0.52, *p* = 0.025) were rated more strongly; knowledge about mold (not covered in the conference) was not significantly different after the conference (*p* = 0.27). Knowledge provided regarding children and environmental hazards was judged to be of great importance (mean 4.85), and was also judged to be sufficient to change practice/policy decision making (mean 4.33).

### 3.2. Discussion

There are two main findings in this manuscript: (1) policy makers in Northwest China appreciate that children are uniquely vulnerable, though additional regulations are needed to account for that vulnerability; and (2) educational interventions can improve policy maker and health care provider knowledge about environmental hazards, including but not limited to air pollution.

There are some limitations that potentially affect the interpretation from study results. The usual caveats about selection bias and external validity to the population of child health providers and policy makers in Northwest PRC apply. Despite a waiver of signed consent, concerns about identifiability with respect to their attitudes may have limited respondent candidness, and there may have been a tendency to give socially appropriate answers, influencing the results. Participation may have also been limited to a subsample of policy makers and providers interested in or more willing to support initiatives to protect children from environmental hazards. In addition, we were only able to evaluate attitudes and beliefs both immediately before and after the conference, and so we cannot evaluate the permanence of changes in attitudes and knowledge that we identified. Assessing validity of self-assessed efficacy is also very difficult, as even basic assessments of children’s environmental health proficiency have not yet been developed.

Due to limited sample size, we only analyzed pre- and post-conference evaluations to compare group-wide attitudes before and after the conference. Future work could assess differential impact of such educational interventions between policy makers and health care providers, or by province. The latter could be of particular interest, especially as the region we studied spans a large geographic area, and diversity within this region also exists in environmental exposures. For example, occupational nickel exposure is a substantial exposure due to the presence of the world’s third largest nickel refinery in Gansu [22], while Qinghai is home to iron, steel and oil industries [23]. Qinghai has multiple large communities living at high altitudes, which may result in confounding and contributing influences on neurodevelopment [24].

While lack of existing research was not identified as a barrier to prevention, authors have previously emphasized the urgent need for studies of chronic air pollution exposures in PRC. The authors also highlighted the modest capacity for translation of knowledge gained from studies from US and other industrialized countries to the PRC population [25]. In this respect, the medical department of the National Natural Science Foundation of China (NNSF), launched in 2010, could support infrastructure development and maintenance over the long term in Northwest PRC.

The policy maker interviews revealed substantially similar results to those previously performed in Mexico [13]. While we acknowledge that qualitative analyses such as these have limits in interpretation, concerns were also raised in Northwest China about threats of economic development as a barrier to proactive protection of children. Yet, there are costs to failing to prevent environmental hazards that can be equally important. For example, childhood lead exposure (chiefly from paint given the recent global eradication of lead in gasoline) contributes $227 billion in lost economic productivity (2.0% of gross domestic product, or GDP) annually as a result of lead-associated decreases in Intelligence Quotient in China [26]. Therefore, educating policy makers about the need to prevent environmental hazards should also include economic data, as implications of environmental hazards for increased health care costs and reduced economic productivity are highly relevant for ministries, especially when they represent a potentially substantial proportion of GDP.

It is true that enhanced knowledge alone does not change policy and enforcement. However, this successful educational intervention could be expanded in scale and scope, potentially by local champions who have received more intensive training, to elicit more concrete policy and regulatory changes. This would follow the model successfully used for children’s environmental health policy champions in the US [27]. Further research could then examine both the effectiveness of the intervention on a larger scale and scope, and utility in improving effective translation of research.

## 4. Conclusions

Policy makers interviewed in Northwest PRC appreciate that children are uniquely vulnerable to the adverse effects of hazardous environmental exposures, though additional regulations are needed to account for that vulnerability. An educational intervention can ameliorate gaps in effective translation of research, improving policy maker and health care provider knowledge about environmental hazards, including but not limited to air pollution. Overall, building knowledge and capacity in CEH should be considered an emerging priority in rapidly developing countries like China, to protect the health and well-being of future generations.
